# The mysterious steps in carcinogenesis: addendum

**DOI:** 10.1038/sj.bjc.6605332

**Published:** 2009-10-13

**Authors:** D Brash, J Cairns

**Affiliations:** 1 Yale Comprehensive Cancer Center, Yale School of Medicine, New Haven, CT 06520-8040, USA; 2 Clinical Trial Service Unit, University of Oxford, Oxford OX3 7LF, UK; 3 105 Alleyn Park, London SE21 8AA, UK

**Sir,**

In the past 100 years, many explanations have been proposed for the process of carcinogenesis but none of them has proved to be totally persuasive. For this reason, we deliberately did not offer a modern synthesis in our review article (Brash and Cairns, 2009). However, in the last few years, thanks to certain experiments, a possible interpretation has emerged, which could be of practical importance.

We now see that all cells (bacterial, yeast and mammalian cells) are more far-sighted than we had imagined. Confronted by stressful or damaging changes in their environment, populations of cells activate a programme that raises their mutation rate for several generations but temporarily masks the mutant phenotypes. This greatly increases the likelihood that some of them will be able to flourish in the new environment.

Two important observations suggest that induction of this ‘stress response’ might be the crucial initiating event in cancer. (1) When cells are exposed to chemical or physical initiators *in vitro*, every cell can be initiated so that it yields transformed descendants, which implies that initiation is the long-term activation of a programme rather than the production of mutations in certain genes (Kennedy *et al*, 1984). (2) Inactivation of one of the genes involved in the stress response protects mice against various experimental cancers (Dai *et al*, 2007).

If the formation of most cancers is initiated by activation of a programme that depends on the interplay of several gene products, then defects in some of these products (although evolutionarily deleterious) might prevent most cancers; therefore, it may be useful to look for polymorphisms that protect against cancer rather than, as has become usual these days, concentrate solely on those that increase the risk. This could not easily be done with humans (whose lifetime risk of cancer is only about 50%), but could be done with mice. Even within inbred strains, mice are known to vary in susceptibility to skin cancer, and only a few generations of selective breeding can produce mice that are largely insusceptible (Boutwell, 1964). So the project would be to look for the genetic changes that accompany such selection and then, if found, study the frequency of changes in the equivalent human genes in relation to the risk of cancer, using the DNA samples that have already been collected for the many studies of genetic susceptibility.
